# Are insecticide-treated bednets more protective against *Plasmodium falciparum *than *Plasmodium vivax-*infected mosquitoes?

**DOI:** 10.1186/1475-2875-5-15

**Published:** 2006-02-21

**Authors:** Moses J Bockarie, Henry Dagoro

**Affiliations:** 1 Papua New Guinea Institute of Medical Research, PO Box 378, Madang, Papua New Guinea; 2 Center for Global Health and Diseases, Case Western Reserve University School of Medicine, 2103 Cornell Rd. Room 4123, Cleveland, Ohio 44104, USA

## Abstract

**Background:**

The outcomes of insecticide-treated bednet (ITN) interventions for malaria control in Papua New Guinea tend to suggest a differential protective effect against *Plasmodium falciparum *and *Plasmodium vivax*. Little is known about the impact of ITNs on the relative abundance of mosquitoes infected with either *P. falciparum *or *P. vivax*. This paper describes the biting cycle of *P. falciparum *and *P. vivax*-infected mosquitoes and the impact of an ITN intervention on the proportion of mosquitoes infected with either parasite species.

**Methods:**

Entomological investigations were performed in East Sepik (ESP) and New Ireland Provinces (NIP) of PNG. Mosquitoes were collected using the all-night (18:00 - 06:00) landing catch and CDC light-trap methods and species specific malaria sporozoite rates were determined by ELISA.

**Results and discussion:**

The distribution of sporozoite positive mosquitoes in three four-hour periods (18:00-22:00, 22:00-02:00 & 02:00-06:00) showed that a higher proportion of *P. vivax*-infected mosquitoes were biting before people retired to bed under the protection of bednets. In the intervention village, the 308 mosquitoes collected before ITNs were introduced included eight (2.0%) *P. falciparum*-positive and four (1.0%) *P. vivax-*positive specimens, giving a parasite ratio of 2:1. The sporozoite rate determined from 908 mosquitoes caught after ITNs were introduced showed a significant decrease for *P. falciparum *(0.7%) and a slight increase for *P. vivax *(1.3%), resulting in a post intervention parasite ratio of 1:2. In the East Sepik Province, where ITNs were not used, *P. falciparum *remained the dominant species in 12 monthly mosquito collections and monthly *P. falciparum*:*P. vivax *formula varied from 8:1 to 1.2:1.

**Conclusion:**

These findings suggest that people sleeping under treated bednets may be more exposed to *P. vivax *than *P. falciparum*-infected mosquitoes before going to sleep under the protection of bednets. This difference in the biting behaviour of mosquitoes infected with different malaria parasites may partly explain the change in the *P. falciparum*:*P. vivax *formula after the introduction of ITNs.

## Background

All four human malaria species are found in Papua New Guinea (PNG), with a predominace of *Plasmodium falciparum *and *Plasmodium vivax *[1]. The outcomes of insecticide-treated bednet (ITN) interventions for malaria control in PNG tend to suggest a differential protective effect against *P. falciparum *and *P. vivax*. Millen [2] working in the coastal areas of Madang Province showed a significant reduction in the prevalence of *P. falciparum *among children between one to four years of age, after treated bednets were introduced, but there was no effect on *P. vivax *prevalence. Graves *et al *[3] who introduced ITNs in different villages in the same province reported a significant reduction in the incidence of *P. falciparum *in children 0–4 years old, 4–10 weeks after the ITNs were introduced; however, they also found no effect on the incidence of *P. vivax*. In the Highlands Region, the *P. falciparum*:*P. vivax *ratio in all age-groups changed from 3.8:1, before ITNs were introduced, to 1:1 six months afterwards [4]. Other phenomena such as *P. vivax *relapses may also lead to a change in the prevalence of *P. vivax *in humans independent of ITN interventions. Relapses are a feature of vivax malaria that may occur 8–10 weeks after a previous attack (short-term relapses) or about 30–40 weeks later (long term relapses). The form and frequency of relapses depend on the infecting strain and occur due to the activation of hypnozoites in liver cells [5].

Little is known about the impact of ITNs on the relative abundance of mosquitoes infected with either *P. falciparum *or *P. vivax*. One study on the biting pattern of *Plasmodium*-infected *Anopheles punctulatus *mosquitoes, the main vector in PNG, suggests a differential feeding behaviour of mosquitoes infected with *P. vivax *and *P. falciparum*, with *P. vivax*-infected mosquitoes having a tendency to bite earlier than *P. falciparum*-infected mosquitoes [6]. If indeed *P. vivax*-positive mosquitoes have a greater tendency to bite earlier than *P. falciparum*-positive ones, people may be more exposed to *P. vivax *than *P. falciparum *before going to sleep under the protection of bednets. This could result in changes in the proportion of people infected with the two species following a bednet intervention. This paper describes the biting cycle of *P. vivax*- and *P. falciparum*-infected mosquitoes and the impact of an ITN intervention on the relative abundance of mosquitoes infected with either parasite species.

## Methods

The impact of ITNs on the parasite ratio in mosquitoes was investigated on Lihir Island in New Ireland Province. The island, about 240 km^2^, is bounded by 70 km of coastline with savannah grassland in the north-east and swampy vegetation with thick mangrove shores in the south-west. The average annual rainfall is about 4,000 mm with no distinct dry and wet seasons. The inhabitants live in coastal villages, usually adjacent to a sandy beach or coral reef. Most of the people of Lihir Island do not normally go to bed before 22:00. In September 1993, the PNG government started the sale of permethrin-treated bednets in all 25 villages on the island, at a subsidized price of US$ 3.00 per net. By October 1993, over 50% of the island population reported that they were sleeping under treated bednets. Pre-treatment blood slides obtained from 471 randomly selected residents (all age-groups) of five indicator villages in September 1993 revealed prevalence rates of 31% for *P. falciparum *and 6.3% for *P. vivax*, hence a parasite formula (*P. falciparum*:*P. vivax*) of 5:1. Follow-up blood slide surveys were not carried out by the Health Department team, but entomological monitoring of the bednet intervention was performed by the Papua New Guinea Institute of Medical Research. Mosquito sampling was conducted on a fly-in fly-out basis as the entomology laboratories were located about 700 km from Lihir island. Sampling could, therefore, only be carried out during four months; one month before and three months after intervention. However, mosquitoes were processed individually to determine infection with malaria parasites.

Mosquitoes were collected using the all-night (18:00 - 06:00) landing catch method and CDC light-traps placed in bedrooms between 18:00 and 06:00. Human biting mosquitoes were caught by two male adults seated outside houses and sporozoite rates were determined for *P. falciparum *and *P. vivax *as described previously [6,7]. Differences in sporozoite rates were evaluated using the Chi-square test. To investigate the extent of variation in monthly sporozoite rates in the *An. punctulatus *complex in the absence of bednets, sporozoite rates were analysed for *P. vivax *and *P. falciparum *for 12 consecutive months (January – December 1997), in the highly malarious village of Yauatong in the East Sepik Province. This village, where less than 3% of the population slept under bednets, has already been described by Bockarie *et al *[6].

## Results and discussion

**Figure 1:**
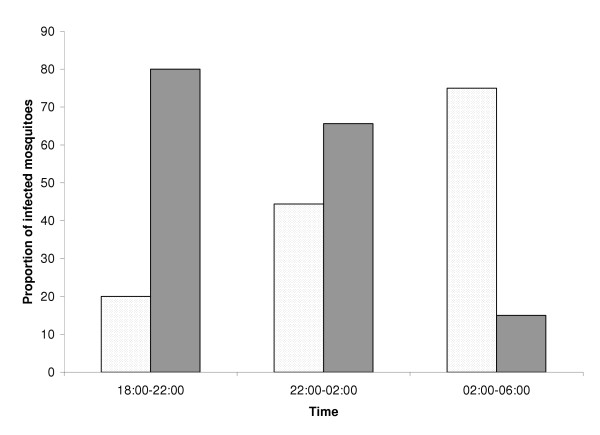
Proportions of *Plasmodium falciparum (shaded bar) *and *P. vivax *(dotted bar) positive *Anopheles farauti *in three four-hour time periods between 18:00 and 06:00, on Lihir Island, Papua New Guinea.

In August 1993, one month before ITNs were introduced on Lihir Island, mosquitoes were collected in the villages of Wurtol and Talis, for four consecutive nights using the all-night landing catch and CDC light-traps methods. Post-intervention collections were carried out using the same methods in October, November and December 1994. All of the 2,209 *Anopheles *mosquitoes collected were morphologically identified as members of the *An. punctulatus *group including *Anopheles. farauti *s.l. (95.5%), *An. punctulatus *(3.8%) and *Anopheles koliensis *(0.6%). A total of 943 mosquitoes were processed from the landing catch and 26 (2.8%) were sporozoite positive including 14 (1.5%) with *P. falciparum *and 12 (1.3%) with *P. vivax*. Mosquitoes with mixed infections were not included in the analysis for this paper. The distribution of sporozoite positive mosquitoes in the three four-hour periods (18:00-22:00, 22:00-02:00 & 02:00-06:00) showed that a higher proportion of infected mosquitoes biting during the pre-bedtime period (18:00-22:00) were infected with *P. vivax *compared to those infected mosquitoes biting later at night (Figure 1). Four *P. vivax *and one *P. falciparum *positive mosquitoes were caught during the pre-bedtime period and the sporozoite rates for this period were 1.6% and 0.4% respectively compared to 1.2% and 1.8% for the rest of the night. Only 7% of the *P. falciparum*-infected mosquitoes were caught during the pre-bedtime period compared to 33% *P. vivax*-infected mosquitoes. However, statistical significance could not be obtained for the difference in the species-specific sporozoite rates for the pre-bedtime period probably because of the low sporozoite rates normally observed for wild caught malaria vectors. Sporozoite rates of the *An. punctulatus *group of mosquitoes seldom exceed 3% [7]. Bockarie and co-workers [6], who processed 4,168 *An. punctulatus *mosquitoes from landing catches in East Sepik Province, also could not establish a statistically significant difference between the sporozoite rates for *P. vivax *and *P. falciparum*during the pre-bed time period despite observing two times more *P. vivax*- than *P. falciparum*-infected mosquitoes during this period. According to Bockarie *et al *[6], the tendency for younger females to bite earlier than older ones may explain the early biting habit of *P. vivax*-infected mosquitoes which on average are younger than *P. falciparum*-infected mosquitoes because of the shorter duration of sporogony for *P. vivax *(seven days) compared to *P. falciparum *(nine days) [5]. They also showed that *An. punctulatus *mosquitoes infected with L1 larvae of *Wuchereria bancrofti *were biting earlier than those infected with L2 and L3 larvae.

**Figure 2:**
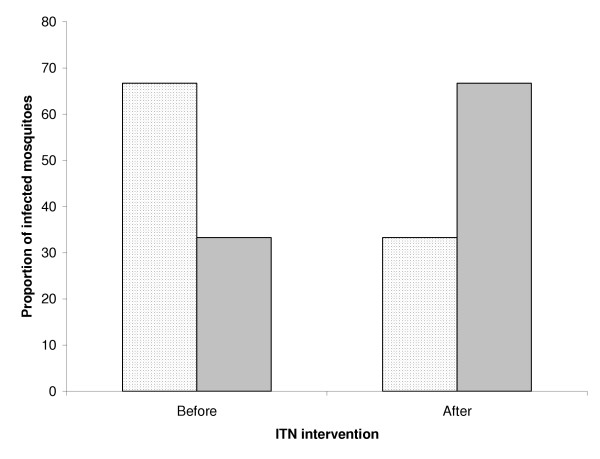
Relative abundance of *Plasmodium falciparum (shaded bar) *and *P. vivax *(dotted bar) positive mosquitoes in mosquito collections performed before and after ITNs were introduced on Lihir island, Papua New Guinea.

A total of 1,302 mosquitoes including light-trap catches were processed to assess the impact of ITNs on parasite ratio. The 394 mosquitoes collected before ITNs were introduced included eight (2.0%) *P. falciparum*-positive and four (1.0%) *P. vivax-*positive specimens giving a parasite ratio of 2:1. The sporozoite rate determined from 908 mosquitoes caught after ITNs were introduced showed a statistically significant (P = 0.027) decrease for *P. falciparum *(0.7%) and a slight increase for *P. vivax *(1.3%) resulting in a post intervention parasite ratio of 1:2 (Figure 2).

However, other phenomena such as relapse may also lead to a change in the prevalence of this species in humans independent of bednet interventions. Relapse is a future of vivax malaria. It represents a reseeding of the bloodstream by dormant parasites ('hypnozoites') contained in the liver. *P. falciparum *does not produce hypnozoites. Relapses may occur 8–10 weeks after a previous attack (short-term relapses) or about 30–40 weeks later (long term relapses). To investigate the extent of variation in sporozoite rates in the *An. punctulatus *complex in the absence of bednets, monthly sporozoite rates were analysed for *P. falciparum *and *P. vivax *for 12 months (January – December 1997) in the highly malarious village of Yauatong in the East Sepik Province. A total of 5,002 mosquitoes were processed from this village, with monthly sample sizes ranging between 118 and 1426 females (Table 1). The annual sporozoite rates for *P. falciparum *and *P. vivax *were 3.1% and 0.6% respectively. Malaria infected mosquitoes were observed in all monthly samples with the exception of September. For the 11 months for which infected mosquitoes were observed, the monthly sporozoite rate for *P. falciparum *was higher than *P. vivax*. Monthly *P. falciparum*:*P. vivax *formula varied from 8:1 to 1.2:1. In the absence of bednets, *P. falciparum *remained the dominant species in monthly mosquito collections suggesting that other factors other than relapse may be responsible for the relative increase in the proportion mosquitoes carrying *P. vivax *following the introduction of ITNs in a community. In a malaria endemic area in India, where *P. vivax *relapse rate ranged from 23% to 44%, the vectors mosquitoes (*Anopheles culicifacies *and *Anopheles stephensi*) remained uninfected during the months (nontransmission season) when relapse rate was highest [10]. It is, therefore, unlikely that the increase in the *P. vivax *infection rate of *An. farauti *observed during this study was predominantly due to relapse.

**Table 1:** *Plasmodium falciparum *and *P. vivax *sporozoite antigen positivity rates and parasite ratios (*P. falciparum*: *P. vivax*) for monthly catches of human-biting *An. punctulatus *mosquitoes in East Sepik Province, Papua New Guinea.

Month	Number processed	Number infected with		PF:PV
			
		*P. falciparum *(%)	*P. vivax *(%)	
Jan	118	2 (1.7)	2 (1.7)	1:1
Feb	324	8 (2.5)	0 (0.0)	0
Mar	1426	48 (3.4)	12 (0.8)	4:1
Apr	326	14 (4.3)	0 (0.0)	0
May	662	6 (0.9)	0 (0.0)	0
Jun	180	14 (7.8)	2 (1.1)	7:1
Jul	398	32 (8.0)	4 (1.0)	8:1
Aug	574	10 (1.7)	8 (1.4)	1.3:1
Sep	219	0 (0.0)	0 (0.0)	0
Oct	278	6 (2.2)	1 (0.4)	6:1
Nov	276	3 (1.1)	2 (0.7)	1.5:1
Dec	221	11 (5.0)	1 (0.45)	11:1
Total	5002	154 (3.1)	32 (0.6)	5:1

## Conclusion

These findings suggest that people sleeping under treated bednets in the Lihir villages may be more exposed to *P. vivax*- than *P. falciparum*-infected mosquitoes before going to sleep under the protection of bednets. This difference in the biting behaviour of mosquitoes infected with different malaria parasites may partly explain the change in the *P. falciparum*:*P. vivax *formula after the introduction of bednets. A similar differential feeding behaviour of mosquitoes infected with the two species have been reported in the East Sepik Province of Papua New Guinea [6]. Other studies in Papua New Guinea have shown that the use of untreated bednets to protect against mosquito bites can also lead to an increase in the proportion of *P. vivax *infections in mosquitoes [8] and humans [4,8]. Recent studies in Madang Province showed that *P. vivax *is becoming more prevalent relative to *P. falciparum *with the increasing use of ITNs in the Province. On Bagabag island, where over 60% of the inhabitants slept under bednets, there was no significant difference (P = 0.129) in the prevalence of *P. falciparum *(19.5%) and *P. vivax *(17.1%) parasites [9]. On the other hand, the prevalence of *P. vivax *(9%) was significantly lower (P < 0.0001) than *P. falciparum *(23%) in Liksul village where less than 15% of the population were sleeping under bednets [11].

## Authors' contributions

MJB designed the study and drafted the manuscript. HD supervised the field work and performed the laboratory analysis.
